# Efficacy of Dose-Escalated Hypofractionated Radiosurgery for Arteriovenous Malformations

**DOI:** 10.7759/cureus.52514

**Published:** 2024-01-18

**Authors:** Sophia N Shah, Sohan S Shah, Praneet Kaki, Sudhakar R Satti, Sunjay A Shah

**Affiliations:** 1 Radiation Oncology, Christiana Care Health System, Newark, USA; 2 Interventional Neuroradiology, Christiana Care Health System, Newark, USA

**Keywords:** fractionated stereotactic radiotherapy, interventional radiology guided embolization, stereotactic radiosurgery (cyberknife®), arterio venous malformations, brain avm obliteration

## Abstract

There is considerable controversy about the management of arteriovenous malformations (AVMs) that are high risk for surgical resection. Stereotactic radiosurgery (SRS) has a reported success rate of less than 50% with unacceptably high rates of radiation necrosis with larger AVM volumes. Neither volume staging nor hypo-fractionated SRS have conclusively been demonstrated to improve results. We hypothesized that the failure of previous hypo-fractionation SRS trials was due to an insufficient biologically effective dose (BED) of radiation. We initiated a pilot study of treating AVM patients with a total dose divided into three or five fractions designed to deliver the equivalent BED of 20 Gy in a single fraction (α/β =3). We performed a retrospective analysis of 37 AVM patients who had a minimum of two years of follow-up or underwent obliteration. Patients were treated with 30 Gy/3 fractions, 33 Gy/3 fractions, or 40 Gy/5 fractions using a CyberKnife device (Accuracy Incorporated, Madison, Wisconsin, United States). The primary endpoint was complete AVM obliteration, determined by MRA imaging. Most obliterations were confirmed with diagnostic cerebral angiography. Secondary endpoints were post-radiosurgery hemorrhage and radiation-related necrosis. Kaplan-Meier analysis was used to determine obliteration rates.

From 2013 to 2021, 37 patients fitting inclusion criteria were identified (62% male, average age at treatment = 48.88 years). Fifteen (41%) patients had prior treatment (surgery, radiosurgery, embolization) for their AVM, 32 (86%) had AVMs in eloquent locations, 17 (46%) had high-risk features, and 14 (38%) experienced AVM rupture prior to treatment. The average modified radiosurgery-based AVM score (mRBAS) was 1.81 (standard deviation (SD)= 0.52), and the mean AVM volume was 6.77 ccs (SD = 6.09). Complete AVM obliteration was achieved in 100% of patients after an average of 26.13 (SD = 14.62) months. The Kaplan-Meier analysis showed AVM obliteration rates at one, two, and three years to be 16.2%, 46.9%, and 81.1%, respectively. Post-operative AVM rupture or hemorrhage occurred in one (2.7%) patient, after nine months. Radiation necrosis occurred in four (11%) patients after an average period of 17.3 (SD =14.7) months. The SRS dose used in this study is the highest BED of any AVM hypofractionation trial in the published literature. This study suggests that dose-escalated hypofractionated radiosurgery can be a successful strategy for AVMs with acceptable long-term complication rates. Further investigation of this treatment regimen should be performed to assess its efficacy.

## Introduction

Arteriovenous malformations (AVMs) of the brain are congenital vascular lesions resulting in abnormal connections between arteries and veins without an intervening capillary bed [1]. Cerebral AVMs have an estimated incidence of 1.34 patients per 100,000 person-years, resulting in a prevalence of 30,000 individuals in the United States [2]. AVMs often manifest with symptoms such as headaches, seizures, and hemorrhages. The annual risk of hemorrhage is 2-4% [3]; however, it is estimated that 80% of untreated AVMs become symptomatic by age 40 [4]. 

Generally accepted interventional treatments include surgical resection, endovascular therapy, and stereotactic radiosurgery (SRS). Conservative management is often recommended for unruptured AVMs based on the results of A Randomized Trial of Unruptured Brain AVMs (ARUBA) [5]. Treatment selection traditionally depends upon the patient’s Spetzler-Martin grade. This score assesses surgical risk based on nidus size, location (eloquent or non-eloquent), and venous drainage (deep or superficial) [6]. Various radiosurgery-specific grading systems have also been used for prognostic purposes. The most accepted is the modified radiosurgery-based AVM score or mRBAS. The mRBAS incorporates AVM volume, patient age, and brain location [7]. Surgery is generally recommended for Spetzler-Martin grade one and two lesions, whereas radiosurgery is more commonly used for smaller lesions that are surgically inoperable. There is no standard accepted treatment for high-risk AVM lesions [8]. 

Stereotactic radiosurgery works by delivering focused radiation to injure the vascular endothelium. This induces the proliferation of smooth muscle cells and extracellular collagen, leading to the obliteration of the AVM nidus [9]. The latency period for obliteration is typically one to three years. It is considered the treatment option with the most generalizability, given that it is capable of treating AVMs that are in eloquent regions considered too risky for endovascular therapy or surgical resection [10]. The gold standard method of stereotactic radiosurgery is the Gamma Knife system, which is typically delivered in one fraction, as it traditionally has required an invasive immobilization frame [11] (newer versions do have the ability to fractionate with a relocatable frame). The typical radiosurgery dose for treating cerebral AVMs is >20 Gy (50% isodose line) in a single fraction [1]. Linear accelerator-based radiosurgery systems have also been used; they do not require the use of an invasive frame, which lent itself to dividing the dose into multiple daily fractions or a hypofractionation strategy [12]. This is a well-established strategy in conventional radiation therapy in order to reduce the risk of late toxicities such as radiation necrosis of the brain. 

The management of high-risk AVMs (mRBAS>1.5) remains controversial with no accepted modality of treatment. Previous high-risk AVM studies have utilized two different radiosurgical methods for the treatment of large AVMs to minimize the risk of radionecrosis: volume-staged stereotactic radiotherapy (VS-SRT) and hypofractionation. VS-SRT involves dividing large AVMs into separate regions, each of which is treated sequentially until the entire AVM is irradiated. In contrast, hypofractionation involves giving multiple consecutive fractionated doses of radiation to the entire AVM volume, typically between three and five fractions [13]. Single fraction SRS has a reported success rate of less than 50% with unacceptably high rates of radiation necrosis with larger AVM volumes [14,15]. Neither VS-SRT nor hypofractionated SRS have conclusively been demonstrated to improve results. We hypothesized that the failure of previous hypo-fractionation SRS trials was due to administering an insufficient biologically effective dose (BED) of radiation. We initiated a retrospective pilot study of treating high-risk patients with an escalated total dose divided into three or five fractions designed to deliver a BED of approximately 150 Gy, roughly equivalent to a dose of 20 Gy in a single fraction (α/β =3).

## Materials and methods

Patient selection

Following Institutional Review Board approval, we performed a retrospective chart review of patients with at least two years of post-radiosurgery radiographic follow-up (or achieved obliteration) who underwent hypofractionated CyberKnife (Accuracy Incorporated, Madison, Wisconsin, United States) SRS for the treatment of their AVM. All patients were treated at the Helen F. Graham Cancer Center and Research Institute at Christiana Care Health Systems in Newark, Delaware, United States, between 2013 and 2021. 

Patient treatment

**Figure 1:**
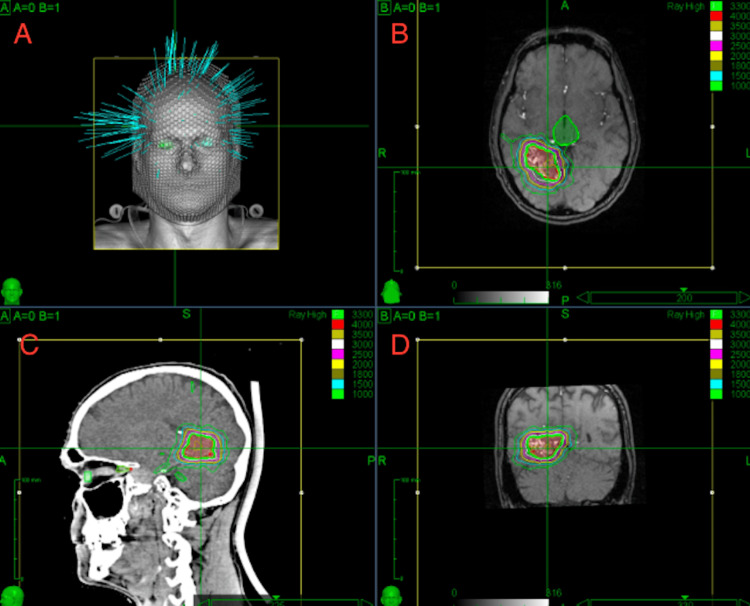
A) Example Cyberknife radiation plan. B) Radiation plan in the axial plane. C) Radiation plan in the sagittal plane. D) Radiation plan in the coronal plane.

Patients were seen by the radiation oncologist after it was determined that their malformation could not be adequately treated with one of the other modes of treatment (endovascular embolization, surgical resection, or medical management alone). Patients underwent both a diagnostic cerebral angiogram as well as MRA of the head. Prior to the CT simulation, patients were immobilized with a thermoplastic mask to limit patient movement. MRA and CT angiography (CTA) images were transferred to the CyberKnife system and fused with the simulation CT scan for planning purposes (Figure 1). AVM target contours were approved by both the neuro-interventional radiologist and radiation oncologist. A highly conformal treatment was designed using the Cyberknife treatment planning software. Initially, patients were treated with the Cyberknife VSI system which was subsequently upgraded to the M6 system. Treatment planning software versions included Accuracy Incorporated's MultiPlan 3.53., Multiplan 5.1.3., and Precision 3.2.0.0. Treatment was delivered using the CyberKnife skull tracking system. 

Patient follow-up

**Figure 2:**
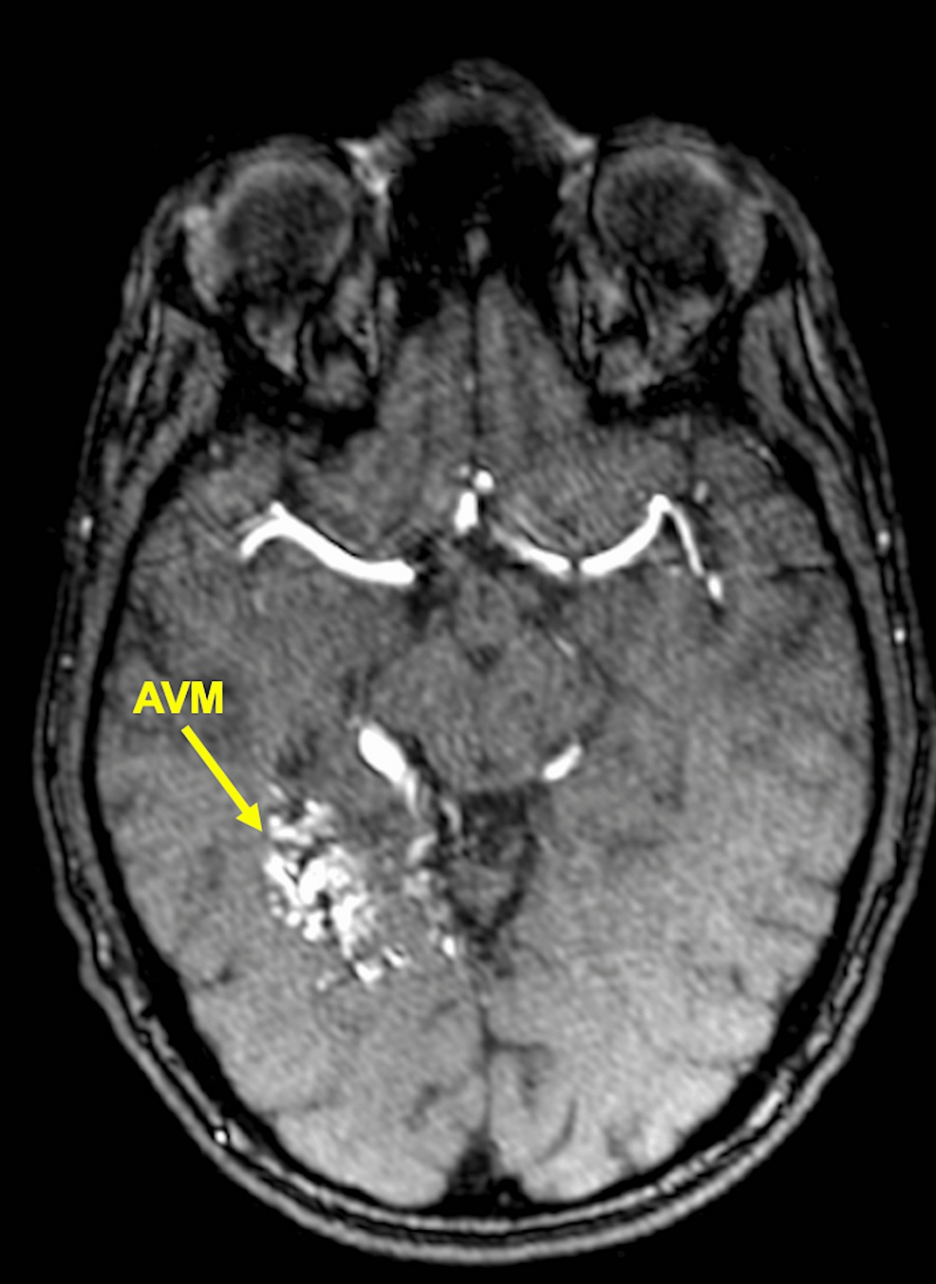
Pre-treatment MRA indicating AVM MRA: magnetic resonance angiography; AVM: arteriovenous malformation

**Figure 3:**
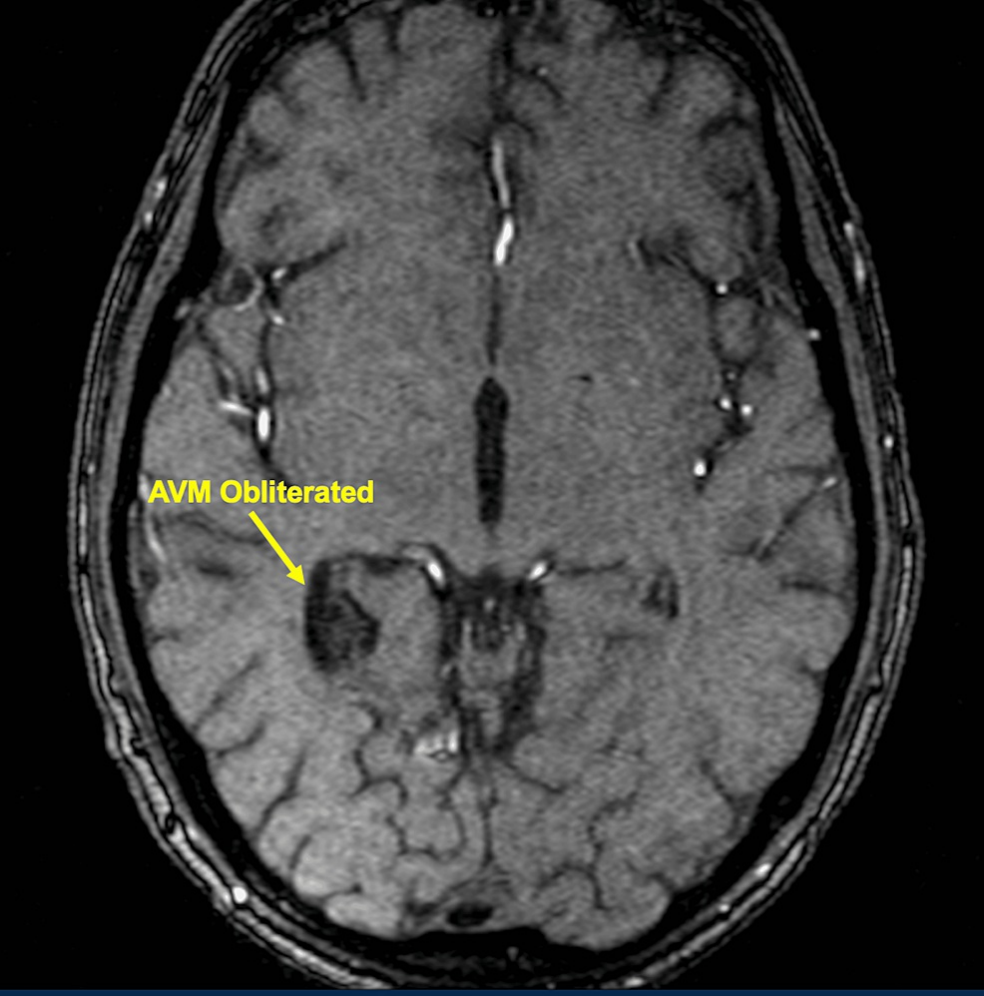
Post-treatment MRA indicating obliteration of the AVM 31 months after Cyberknife* treatment MRA: magnetic resonance angiography; AVM: arteriovenous malformation *Accuracy Incorporated, Madison, Wisconsin, United States

**Figure 4:**
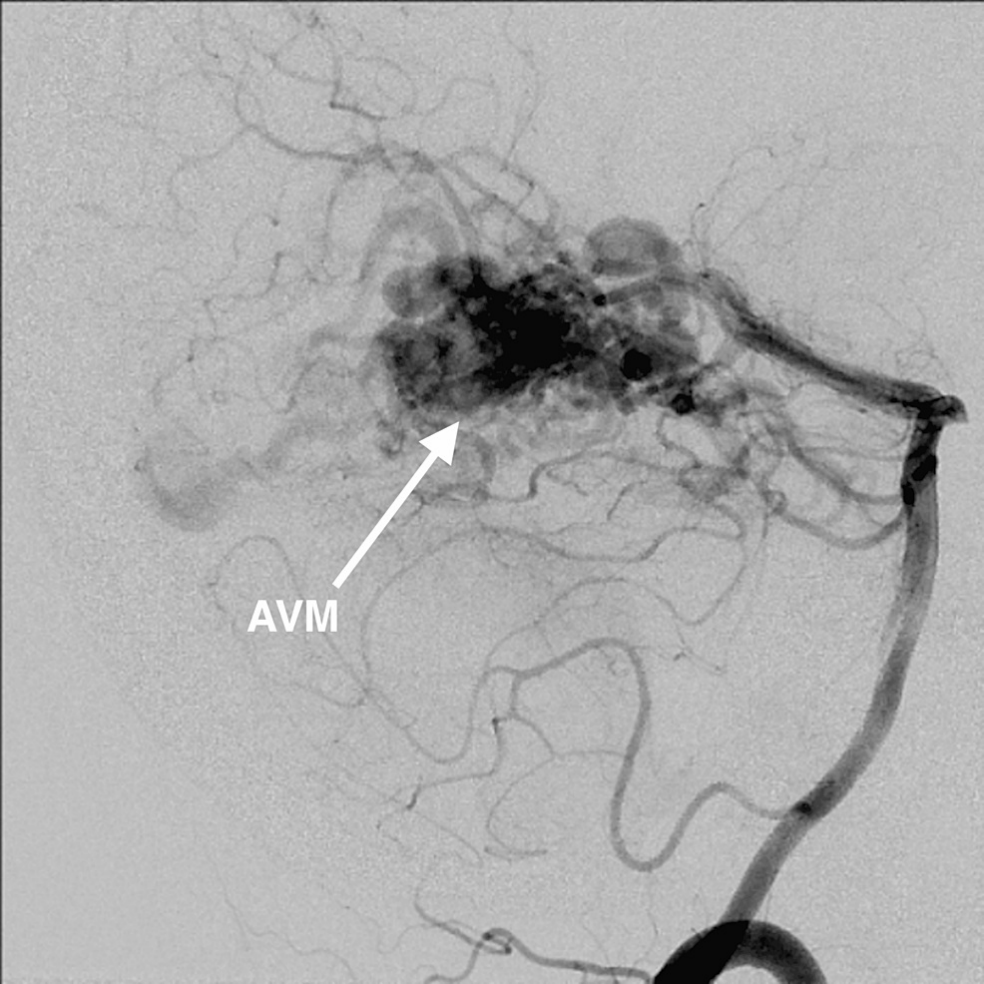
Pre-treatment cerebral diagnostic angiogram indicating AVM AVM: arteriovenous malformation

**Figure 5:**
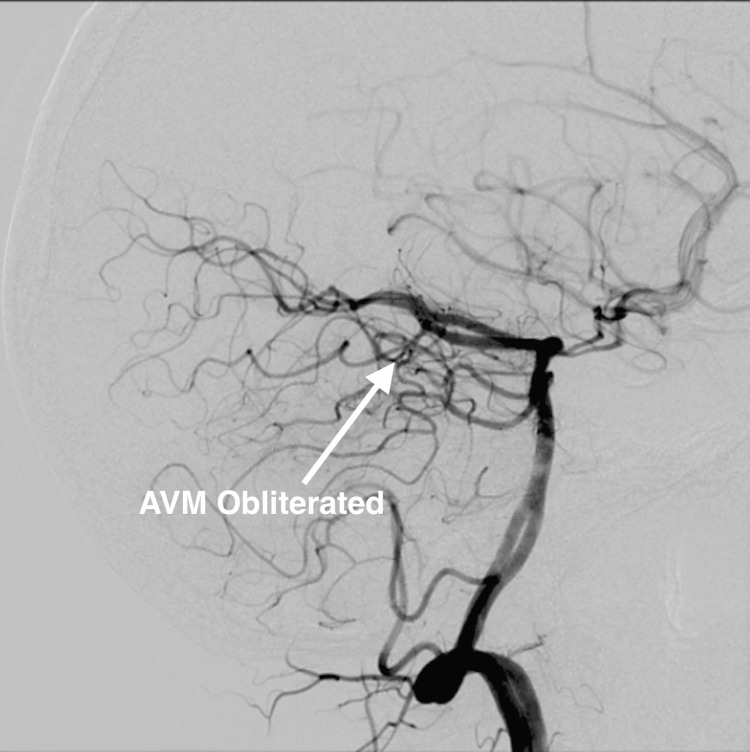
Post-treatment cerebral diagnostic angiogram indicating obliteration of the AVM 27 months after Cyberknife* treatment AVM: arteriovenous malformation *Accuracy Incorporated, Madison, Wisconsin, United States

One year after treatment, patients underwent MRA or CTA imaging to determine if there was a residual nidus or if the AVM had been obliterated (Figures 2, 3). If, however, residual AVM was visualized, the patient would return for a six-month to one-year follow-up and undergo the same imaging protocol. If no residual nidus was visualized, the patient was recommended to undergo conventional cerebral diagnostic angiography to confirm that obliteration had occurred (Figures 4, 5). The mean follow-up was 26.2 months. 

Outcome measures

The primary outcome measures were AVM obliteration and the incidence of radionecrosis following SRS. Obliteration was defined by a greater than 95% reduction in AVM volume. More than 80% of cases were confirmed by diagnostic cerebral angiography. Radionecrosis was detected by MRI. Radiation necrosis was defined as contrast enhancement MRI associated with T2 changes and persistent symptoms requiring steroids. Independent variables that we characterized included age, sex, prior surgical resection/embolization, time to AVM obliteration, presence in an eloquent region, high-risk features, AVM rupture prior to SRS, rupture or hemorrhage post-SRS, radionecrosis following treatment, maximum AVM diameter, total AVM volume, the Spetzler-Martin score, and the mRBAS. These variables were also analyzed to identify predictors of post-treatment radionecrosis. 

Statistical analysis

Mean (standard deviation) was used to present continuous variables, and frequency (percentage) was used for categorical variables. Kaplan-Meier survival curves were used to depict obliteration and radionecrosis rates following radiosurgery. Univariate and multivariate analyses were used to identify predictors of post-operative radionecrosis. Statistical significance was defined as a p-value less than 0.05. All statistical analysis was performed using R Studio, version: 2023.03.0+386 (Posit PBC, Boston, Massachusetts, United States).

## Results

Patients, AVM, and SRS characteristics

A total of 45 patients underwent hypo-fractionated radiosurgery for large AVMs between 2013 and 2021. A total of 37 patients had a minimum of two years of radiographic follow-up and were analyzed in the study. One patient was excluded from our analytic cohort because an incorrect location was targeted for SRS. This patient was subsequently treated with SRS to the correct target but did not have two years of follow-up. Twenty-three (62%) patients were male (Table 1). The average age of the cohort at the time of the radiosurgery was 48.9 years (SD = 13.5). Fifteen (43%) patients underwent surgery, radiosurgery, or embolization for their AVMs prior to receiving hypo-fractionated radiosurgery. Fourteen (38%) patients experienced an AVM rupture prior to treatment. Thirty-two (86%) patients had AVMs deemed to be located in eloquent locations, and 17 (49%) presented with high-risk features. The average maximal AVM diameter was 2.36 cm (SD=1.28) and the AVM volume was 6.77 cm^3^ (6.09). The mean Spetzler-Martin grade was 2.86 (SD=0.79) and the median was 3. The mean mRBAS score was 1.81 (SD=0.52).

**Table 1:** Demographic and AVM characteristics ^1^Values indicate the number of patients (%) unless otherwise indicated AVM: arteriovenous malformation; mRBAS: modified radiosurgery-based AVM score mRBAS = 0.1 × volume (cm3) + 0.02 × age (years) + 0.5 × location (deep location: basal ganglia, thalamus, or brainstem = 1, else location = 0)

Characteristic	N=37^1 ^
Age, years (SD)	48.88 (13.46)
Sex	
Male	23 (62%)
Female	14 (38%)
Treatment regimen	
10x3	14 (38%)
11x3	17 (46%)
8x5	6 (16%)
Prior surgery, radiosurgery, or embolization	15 (41%)
Eloquent location	32 (86%)
High risk features (venous stenosis or aneurysm)	17 (46%)
Rupture prior to treatment	14 (38%)
Spetzler Martin grade	2.86 (0.79)
AVM mRBAS score	1.81 (0.52)
AVM size (ccs)	6.77 (6.09)
AVM maximal diameter (cm)	2.36 (1.28)

Outcomes

**Figure 6:**
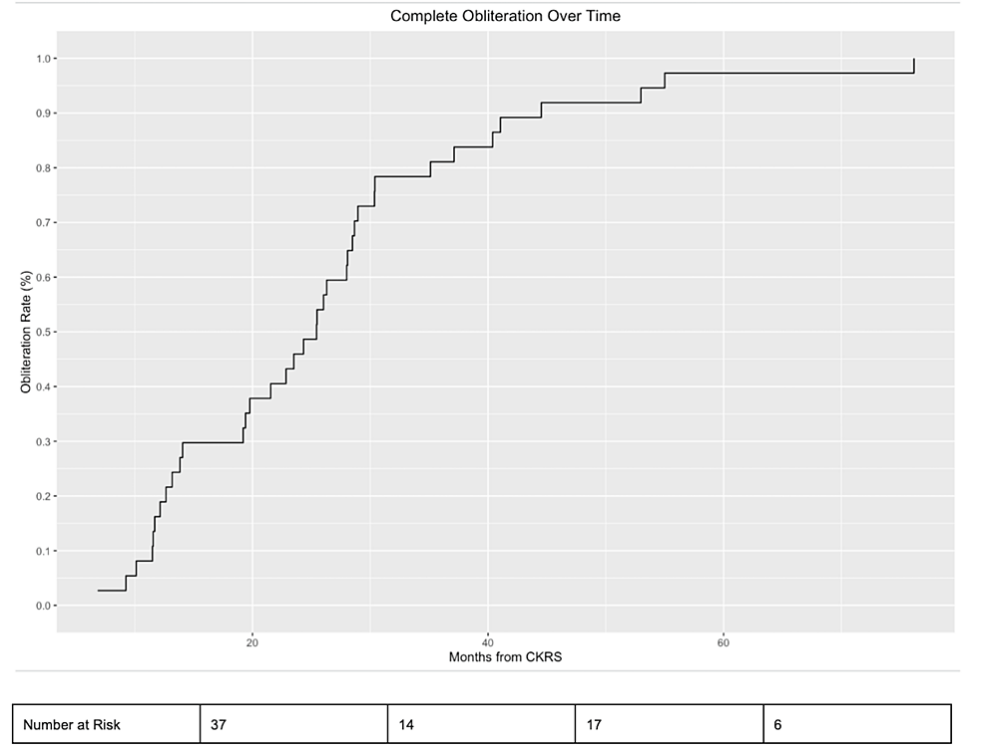
Complete obliteration over time CKRS = Cyberknife radiosurgery

All 37 (100%) patients in this study experienced complete AVM obliteration, after an average of 26.2 (SD=14.6) months following hypo-fractionated SRS. One patient underwent retreatment for residual AVM two years after initial hypofractionated treatment and achieved obliteration. The Kaplan-Meier curve for obliteration is depicted in Figure 6. Obliteration probabilities at one-, two-, and 3-year follow-ups were 16.2%, 46.9%, and 81.1%, respectively. Post-operative AVM rupture or hemorrhage occurred in one (2.7%) patient, after nine months. 

**Figure 7:**
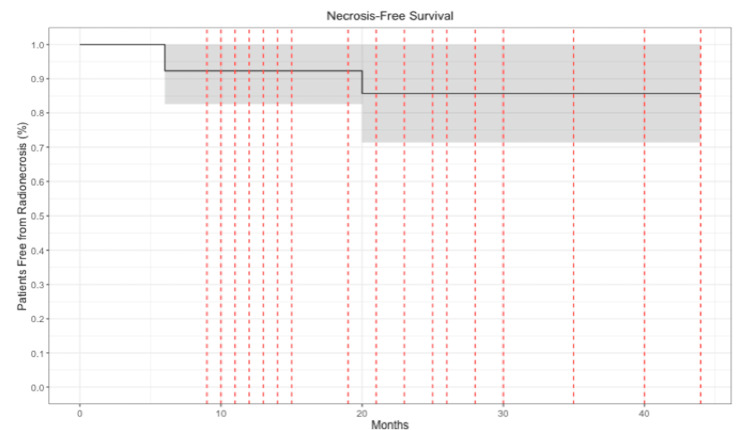
Necrosis-free survival

The Cyberknife treatment had minimal acute toxicity. The patients were routinely pre-medicated with steroids. Three patients had mild headaches, but there were no severe headache episodes or seizures. The only late toxicity was radiation necrosis. Radiation necrosis occurred in four (11%) patients after an average period of 17.3 (SD=14.7) months (Table 2). Radionecrosis-free survival probabilities after one, two, and three years following SRS were 94.6%, 90.5%, and 90.5%, respectively (Figure 7). Univariate logistic regression analysis was performed to identify predictors of radionecrosis following SRS. Large maximal AVM diameter (≥ 3.0 cm) was the only factor associated with a significantly higher risk of experiencing radionecrosis (odds ratio (OR) = 6.86, 95% CI =1.03, 48.4, p=0.046).

**Table 2:** Clinical outcomes following CKRS ^1^Values indicate the number of patients (%) unless otherwise indicated AVM: arteriovenous malformation; CKRS: Cyberknife radiosurgery

Characteristics	N=37^1 ^
Experienced AVM obliteration	37 (100%)
Months to obliteration	26.13 (14.62)
Rupture/hemorrhage post-treatment	1 (2.7%)
Months to post-op rupture	9 (NA)
Radiation necrosis post-treatment	4 (11%)
Months to radiation necrosis	17.25 (14.73)

## Discussion

The three main modalities for treating AVMs are radiosurgery, microsurgery, and embolization. Radiosurgery has been shown to be effective for small, compact AVMs (<4 mL), resulting in an 85-100% obliteration rate [16,17]. Surgery and embolization are effective as well with obliteration rates of 94-98% and 76% [18], respectively. However, for high-risk AVMs, there is no standard accepted method of treatment. The results with radiosurgery have been suboptimal. A large meta-analysis of AVM patients treated with single-stage Gamma Knife radiosurgery (GKRS) reported AVM obliteration rates of 32.4% for Spetzler-Martin Grade IV/V AVMs [19]. Starke et al. reported on a cohort of 2236 AVM patients from eight medical centers participating in the International Gamma Knife Research Foundation treated from 1988 to 2013. For high AVMs (mRBAS>1.5), the single fraction radiosurgery favorable outcome rate was 45% [20]. Many different radiosurgical approaches have been tried to improve the high-risk AVMs, such as pre-SRS embolization, VS-SRT, and hypofractionation with minimal success. 

Studies have previously shown that a minimum BED of radiation is required to induce high rates of obliteration. A single fraction prescribed dose of 20 Gy and BED of 153.3 Gy is the established recommendation for AVM treatment [1]. Unfortunately, the risk of radiation necrosis starts increasing once the target size is larger than 2.5 cm and the BEDs of the prescribed doses are typically decreased. In the past, linear accelerator-based hypofractionation studies treating high-risk AVMs have used relatively low BED hypofractionation schemes, and the reporting of radiographic versus symptomatic radionecrosis has been inconsistent. Sparks et al. reported the treatment results of patients with AVMs >3 cm treated with a hypofractionated regime of either 25 Gy/5 fractions, 30 Gy/5 fractions, or 30 Gy/6 fractions. Complete obliteration was achieved in only 11.9% of patients; however, the high dose 30 Gy/5 fraction arm did result in a significantly higher obliteration rate of 41% [21]. Aoyama et al. reported a 53% three-year obliteration rate without any radionecrosis using a dose of 28 Gy/4 fractions prescribed to the periphery of the lesion [22]. Chen et al. described 35 patients treated with 35 Gy/5 fractions or 28 Gy/4 fractions. This resulted in a 74% obliteration rate but with a 25.7% rate of symptomatic radiation necrosis [23]. Veznedaroglu et al. described 23 large AVMs treated with 30 Gy/6 fractions with an obliteration rate of 22%. Seven AVMs treated with 42 Gy/6 fractions had a significantly higher obliteration rate of 83%; however, six out of the seven patients developed T2-weighted changes on MRI [24]. These results were not convincing to establish hypofractionated SRS as a standard treatment technique for high-risk AVMs (Table 3). 

**Table 3:** Literature review of retrospective studies evaluating stereotactic radiosurgery BED: biologically effective dose

Series	Number of patients treated	Total dose (Gy)/number of fractions	BED (a/b=3) (Gy)	Single fraction equivalent (Gy)	Obliteration rate (%)	Radiation necrosis rate (%)	Hemorrhage rate (%)
Shah et al., 2023 (current study)	37	30/3	130	18.3	100	11	2.7
		33/3	154	20.0			
		40 /5	146.7	19.5			
Sparks et al., 2019 [21]	42	25/5	66.7	12.7	11.9	11.9	4.8
		30/5	90.0	15.0			
		30/6	80.0	14.1			
Chen et al., 2016 [23]	35	35/5	116.7	17.3	74	25.7	5.7
		28/4	93.3	15.3			
Veznedaroglu et al., 2008 [24]	7	42/6	140.0	19.1	83	42.8	
	23	30/6	80.0	14.1	22	13.0	
Aoyama et al., 2001 [22]	26	28/4	93.3	15.3	53 (three-year actuarial rate)	0	8

Our hypothesis was that previous linear accelerator hypofractionation trials did not achieve high obliteration rates due to the insufficient BED of their radiation dose fractionation schemes. We felt that we could use the Cyberknife device to safely escalate the dose of hypofractionated SRS to the same BED (approximately 150 Gy using α/β=3) as the established 20 Gy threshold dose used in single fraction treatment. We prescribed dose escalated hypofractionation for patients who felt to be at high risk for complications either because of eloquent location, irregular contour, or AVM diameter. Cyberknife has the advantage of delivering tightly conformal plans with a large number of beam angles similar to the fixed source Gamma Knife but with a linear accelerator platform allowing hypofractionation. We initially treated patients with a prescribed dose of 30 Gy/3 fractions for medium-sized lesions and 40 Gy/5 fractions for larger volumes. After we felt comfortable that the 30 Gy/3 fraction was well tolerated, we increased the dose for medium-sized lesions to 33 Gy/3 fractions. 

With a median follow-up of 26.2 months, all 37 patients achieved obliteration defined as >95% obliteration of the nidus on MRA or CTA scan. The mean time to obliteration was 26.1 months. More than 80% of the patients had confirmation of obliteration by diagnostic cerebral angiography. Despite the lack of cerebral angiography confirmation for all patients, MRA has been shown to be an accurate measure of obliteration after SRS with an extremely low risk of hemorrhage [20]. The presumption is that the slow flow rate and low pressure of minimally shunting residual AVM after SRS are unlikely to result in a hemorrhage. Despite using an escalated radiation dose, the risk of symptomatic radiation necrosis was acceptable at four out of 37 patients (11%) occurring at an average time of 17.3 months. Only one patient (2.7%) suffered a hemorrhage after the radiosurgical treatment. 

The radiosurgery scoring systems were developed in order to aid the determination of prognosis and allow cross-trial comparison of radiosurgical series. A total of 27 out of 37 patients (70.3%) in our study were considered to have high-risk AVMs, defined as an mRBAS of 1.5 or greater. A favorable outcome, defined as AVM obliteration without posttreatment hemorrhage or permanent radiation-related complication, occurred in 22 out of 26 (84.6%) of these patients. These results compare well with those of a large multicenter retrospective cohort of 2236 AVM patients treated with single fraction GKRS, which found the favorable outcome rate was 45% in similarly defined high-risk patients [20]. In our study, 100% of 27 high-risk AVMs, including those with high Spetzler-Martin grades, were obliterated. In contrast, Chen et al. reported a 44% obliteration rate with 35 Gy/5 fractions or 28 Gy/4 fractions in 18 patients with mRBAS of >1.5 [23].

On univariate analysis (Table 4), only AVM diameter was associated with radionecrosis. The one patient who experienced radionecrosis and did not have an AVM diameter greater than 3 cm underwent a repeat CKRS treatment (21 Gy in one fraction) for residual AVM two years following their initial hypofractionated CKRS regimen. The other three patients had AVM diameters greater than 3 cm and were treated with a three-fraction schedule. Based on these results, our current paradigm is to treat AVMs with a diameter less than 1.5 cm with 21 Gy in one fraction, diameters between 1.5 and 3.0 cm with 33 Gy in three fractions, and those with a diameter greater than 3.0 cm with 40 Gy in five fractions. We wait at least four years before repeating SRS in the absence of a geographic miss. Only two (40%) patients experiencing post-radiosurgery complications had a high-risk Spetzler-Martin grade of four or five. The AVM diameter was more effective at risk-stratifying AVM patients treated with radiosurgery compared to the Spetzler-Martin or mRBAS grades. 

**Table 4:** Univariate and multivariate analyses of outcomes for patients at the final follow-up HR: hazard ratio; mRBAS = modified radiosurgery-based arteriovenous malformation (AVM) scale

		Univariate			Multivariate	
Predictive factor	HR	95% CI	P-value	HR	95% CI	P-value
Age, years	0.96	0.88 - 1.04	0.327	0.97	0.79 - 1.24	0.076
Is male	0.571	0.0618 - 5.26	0.60	0.307	0.002 - 16.3	0.61
Prior rupture	0.51	0.02 - 4.51	0.58	NA	NA	NA
Max diameter	6.86	1.03 - 48.4	0.046	77784.0	3.45 - 1.17e+21	0.198
Volume	1.04	0.86 - 1.21	0.67	0.249	2.14e-3 – 0.815	0.218
High-risk features	1.07	0.12 - 9.83	9.52	0.08	6.67e-8 – 9.27	0.54
Spetzler-Martin	1.5	0.07 - 14.3	0.744	NA	NA	NA
mRBAS	1.3	0.15 - 28.2	0.83	NA	NA	NA

Our results represent a marked improvement in favorable outcomes for high-risk AVMs versus previous reports in the literature. The Cyberknife system provides tight conformality in the treatment of irregular-shaped lesions in conjunction with the ability to hypofractionate treatment. Whether these favorable results can be replicated on other SRS platforms is unknown. Although the literature shows similar conformality between various commonly used radiosurgical platforms [25], it is possible that Cyberknife’s advantages allowed us to safely administer a high BED with a low risk of late toxicity. On the other hand, our patients were treated relatively recently compared to the historical literature and it is likely that continuous improvements in target delineation, plan conformality, and treatment delivery have occurred in all SRS platforms due to technical advances over the last twenty years.

## Conclusions

Dose-escalated hypofractionated SRS resulted in a 100% AVM obliteration rate in 37 patients. The rate of radiation necrosis was 11%. High-risk lesions defined as mRBAS score >1.5 had an 84.6% favorable outcome rate compared to a historical rate of less than 50%. The SRS dose used in this study has the highest BED of any AVM hypofractionation trial in the published literature. This study suggests that dose-escalated hypofractionated radiosurgery can be a successful strategy for high-risk AVMs with acceptable long-term complication rates. Further investigation of this treatment regimen in a multicenter trial should be performed to assess its efficacy.
